# Exploring the Role of Social Networks in Facilitating Health Service Access Among Low-Income Women in the Philippines: A Qualitative Study

**DOI:** 10.1177/11786329211068916

**Published:** 2022-01-20

**Authors:** Kathy Luu, Laura Jane Brubacher, Lincoln L Lau, Jennifer A Liu, Warren Dodd

**Affiliations:** 1 School of Public Health Sciences, University of Waterloo, Waterloo, ON, Canada; 2 International Care Ministries, Manila, Philippines; 3 Dalla Lana School of Public Health, University of Toronto, Toronto, ON, Canada; 4 Department of Anthropology, University of Waterloo, Waterloo, ON, Canada

**Keywords:** Health care access, gender, poverty, social networks, universal health coverage

## Abstract

Despite efforts to implement universal health care coverage (UHC) in the Philippines, income poor households continue to face barriers to health care access and use. In light of recent UHC legislation, the aim of this study was to explore how gender and social networks shape health care access and use among women experiencing poverty in Negros Occidental, Philippines. Semi-structured interviews were conducted with women (n = 35) and health care providers (n = 15). Descriptive statistical analyses were performed to report demographic information. Interview data were analyzed thematically using a hybrid deductive-inductive approach and guided by the Patient-Centred Access to Health Care framework. Women’s decisions regarding health care access were influenced by their perceptions of illness severity, their trust in health care facilities, and their available financial resources. Experiences of health care use were shaped by interactions with health professionals, resource availability at facilities, health care costs, and health insurance acquisition. Women drew upon social networks throughout their lifespan for social and financial support to facilitate healthcare access and use. These findings indicate that social networks may be an important complement to formal supports (eg, UHC) in improving access to health care for women experiencing poverty in the Philippines.

## Background

Social networks play an important role in influencing health outcomes and facilitating health care access. Women, in particular, may draw upon family or broader community networks for health information, resources, and social support.^1-5^ Not only can strong social ties facilitate women’s emotional and psychological well-being, but they can also promote access to health care services through, for instance, the provision of child care or other financial supports.^6-11^ These supports may be especially significant in contexts where gender inequalities and gender-based norms amplify barriers to health care access.^12-14^ Despite the known benefits of social networks to health and health care access, catastrophic health expenditure is one such persistent barrier to health care access and use among women in South East Asia.^15,16^ A patchwork of Universal Health Coverage (UHC) schemes have been implemented across South East Asian countries in recent years with the aim of eliminating out-of-pocket payment for health services, reducing the incidence of catastrophic health expenditure, and facilitating equitable health care access for women.^16^ However, for women living in poverty, additional indirect and opportunity costs associated with health care may deter care-seeking behavior, in spite of UHC policies.^16-20^

In 2018, close to 17% of the population in the Philippines lived under the national poverty line.^21^ The possibility of catastrophic health expenditure has been shown to be a substantial deterrent to health-seeking behavior in the Philippines, especially among individuals experiencing poverty.^22^ Importantly, underlying gender inequality and gender-based norms may also exacerbate barriers to health care access among women in the Philippines, particularly those who hold caregiving roles within their households and feel pressured to conform to “traditional” female norms.^23,24^ Young women disproportionately serve in a caregiving role compared to young men^24,25^ and, as a result, may not want to be perceived as a household financial burden when health problems arise.^26,27^ Thus, women may avoid seeking necessary health care or alter their desired health behavior in order to attempt to mitigate these financial pressures.^28-30^

Recent health reforms in the Philippines have the potential to improve women’s health care access and use, particularly among individuals experiencing poverty. Building on decades of incremental health policy changes,^31^ the Universal Health Coverage (UHC) Bill was signed into law in February 2019. A key feature of this new legislation was the enrollment of all citizens, regardless of socioeconomic status, into the PhilHealth (national health insurance) program.^32^ While intended to eliminate direct, point-of-care health care costs for all citizens, initial reports indicate that the benefits of this UHC reform (and prior policy reforms) have not adequately addressed the needs of women experiencing poverty.^17,18^

In the context of this changing health policy landscape in the Philippines, it is important to investigate the barriers that women experiencing poverty continue to face in accessing health care. While UHC legislation is intended to address direct health care costs, women experiencing poverty may face additional barriers to health care access beyond direct health care costs. Previous research has highlighted the potential role of social networks in influencing health behaviors and outcomes in the Philippines; however, there is a need to further interrogate the role of social networks in facilitating health care access among women experiencing poverty, especially in light of recent health policy reforms. In addition, given preliminary reports that inequities exist in the roll-out of UHC in the Philippines, there is a need to consider how different socio-demographic factors may shape the degree of support, health-related knowledge, and resources available to women experiencing poverty. Thus, the objectives of this study were (1) to explore different factors that influence health care access and use among women at different life stages who experience poverty in the Philippines; and (2) to examine the potential role of social networks in facilitating health care access among women at different life stages who experience poverty in the Philippines.

## Methods

### Theoretical framework

This study was guided by the Patient-Centred Access to Health Care (PCAHC) framework, which considers how multilevel perspectives influence the determinants of health care supply, demand, and access.^33^ The PCAHC framework is appropriate for this study as it interrogates the complex factors influencing health care access, and includes both health systems and population contexts in its analytical framing.^34^ Five dimensions of health care accessibility are captured in this framework: approachability, acceptability, availability and accommodation, affordability, and appropriateness.^33,35,36^ In this study, the PCAHC framework was adapted to inform the semi-structured interview questions used during the data collection phase, and to create the deductive codes and themes during the data analysis stage. Recognizing that health care access, needs, and decision-making may shift across one’s lifespan,^37-39^ we also created age cohorts of participants to further structure our analysis. This approach was used to more intentionally examine possible interactions between age, social networks, and health care access among participants. In addition, this analytical decision was made to augment the PCAHC framework, which does not explicitly consider life stage as a cross-cutting determinant of health care access and use.

### Study area

This study was conducted in and around Bacolod City, which is the capital city of the province of Negros Occidental, Philippines. In total, data were collected in 7 *barangays* (A barangay refers to the smallest political unit in the Philippines. Barangays are comprised of puroks which are smaller zones that are informally divided and considered at the sub-village level.^40^) inclusive of urban, peri-urban, and rural settings. Despite its high level of urbanization, availability of public services, and economic opportunities, 22.6% of households in Bacolod City have an income below the country’s poverty threshold.^41^ Health care is delivered in and around Bacolod City through Rural Health Units which provide primary health care for rural communities in the Philippines. Each Rural Health Unit is responsible for multiple *Barangay* Health Stations that deliver services to catchment areas.^31^ Within Bacolod City, there are 29 *Barangay* Health Stations, 1 government hospital, and 6 private hospitals serving the population.^41^

### Partnership

We collaborated with International Care Ministries (ICM), a Philippines-based non-governmental organization, to conduct this study. ICM works exclusively with ultrapoor households (defined by the organization as a household living on less than $0.50 USD or 22 Philippine Pesos per person per day) to provide health and livelihood interventions. At the time of data collection, ICM operated across 10 bases in 21 provinces across the Visayas and Mindanao, Philippines (including Negros Occidental). ICM’s core strategic program is called *Transform*, which is a 15-week community-based education intervention that includes livelihood support and health promotion. Each *Transform* program recruits approximately 30 individuals at a time. ICM granted institutional support for this project, informed the development of data collection tools, and guided participant recruitment.

### Study participants and recruitment

Participants were purposively selected due to their location (proximity to Bacolod City), and participation in ICM programming (*Transform*), which provided a homogenous group in terms of socioeconomic characteristics. Participants were 18 years of age or older, current *Transform* participants, and accessed and used health services in the region. In addition, several health care providers (including physicians, midwives, and *barangay* health workers) were interviewed to provide additional perspectives and context about service interactions and other structural factors that may influence women’s health care access in the study area.

### Data collection

Data was collected in May 2019 through face-to-face, semi-structured interviews and interpretation assistance. Interviews were conducted post-UHC legislation, though participants reflected on both previous (pre-legislation) and current (post-legislation) experiences with health care access and use. Four data collection tools were developed for this project: 2 questionnaires (for participants and healthcare providers, respectively) and 2 semi-structured interview guides that explored (1) participants’ experiences of accessing and using health care services and (2) health care provider reflections on delivering care to women experiencing poverty. Questionnaires and interviews were conducted in either Hiligaynon or English based on the preferred language of the participant, with data collection co-facilitated by a member of the research team and a trained interpreter. The choice of having real-time language interpretation was made to reduce the need to refine interpretation in subsequent stages of the study.^42^ All interviews were audio recorded and lasted between 30 minutes and 1 hour (mean interview duration = 45.7 minutes).

### Analysis strategy

For quantitative data, descriptive statistical analysis was performed to report basic demographic data on participants from the questionnaires. For qualitative data, audio files were transcribed, with non-English portions of interviews interpreted and transcribed prior to analysis. To examine possible interactions between age, social networks, and health care access, participants were divided into 4 cohorts based on age and in correspondence with distinct life stages (18-30 years; 31-45 years; 46-59 years; 60 years and older). Demographic data were imported into QSR NVivo 12© software and assigned to the corresponding interview transcript to generate cases and classifications. This process was completed to analyze the narratives of participants while remaining aware of each participant’s assigned age cohort and other demographic characteristics.

An iterative process using the PCAHC framework and a modified hybrid deductive-inductive thematic analysis was applied to the interview data to identify and explore themes.^43^ Coding was informed by the research objectives, the interview guide, and elements from the PCAHC framework. During initial coding, deductive codes were identified, classified, and assigned to segments of data.^43,44^ Subsequently, an inductive thematic approach was employed to facilitate a more nuanced analysis of participants’ perspectives and experiences and to integrate provider perspectives into the analysis.^43,44^ Then, the connections among the inductive and deductive codes were identified; codes were merged, re-arranged, and consolidated, as necessary, then re-applied to the data; and emergent themes were identified.^45^

### Ethical considerations

This study was approved by the Research Ethics Board at the University of Waterloo (ORE#40797). Prior to data collection, a description of the study was provided to all participants and written or oral informed consent was obtained. As some participants had a low level of literacy, the option of oral informed consent was provided to participants in accordance with our ethics protocol.

## Results

### Demographic characteristics of participants

Overall, 35 female participants (age range: 18-75 years) were interviewed from 7 *barangays* in and around Bacolod City, Negros Occidental. Across the 4 age cohorts, 10 women were in the youngest cohort (18-30 years), 11 women were in the second youngest cohort (31-45 years), 9 women were in the second oldest cohort (46-59 years), and 5 women were in the oldest cohort (60 years and older).

Twenty-four participants (68.6%) reported that they were PhilHealth beneficiaries. The majority of these participants were married (85.7%), did not work outside their household (60.0%), and had achieved a secondary school education (60.0%) (see Table 1). Women from the youngest age cohort (18-30 years) were less likely to be enrolled in the *Pantawid Pamilyang Pilipino* Program (4Ps; a national conditional cash transfer program) compared to the 3 older age cohorts. Women from the youngest age cohort also attained a higher level of education (often secondary school or college-level education) and had smaller household sizes compared to women in the 3 older age cohorts.

**Table 1.** Demographic characteristics of participants in Negros Occidental, Philippines (n = 35).

	Frequency (%)
Age cohort	
18-30 y	10 (28.6%)
31-45 y	11 (31.4%)
46-59 y	9 (25.7%)
60 y and older	5 (14.3%)
Educational attainment	
No school	1 (2.9%)
Some primary school	12 (34.3%)
Some secondary school	21 (60.0%)
Some college or vocational school	1 (2.9%)
Marital status	
Married	30 (85.7%)
Widow	2 (5.7%)
Single	2 (5.7%)
Common law	1 (2.9%)
Occupation	
No employment outside of the home	21 (60.0%)
Farm labor	5 (14.3%)
Other^a^	9 (25.7%)
Household members	
0-5 members	16 (45.7%)
6-10 members	17 (48.6%)
11-15 members	2 (5.7%)
PhilHealth status	
Beneficiary	24 (68.6%)
Non-beneficiary	9 (25.7%)
Unknown	2 (5.7%)
4Ps status^b^	
Beneficiary	14 (40.0%)
Non-beneficiary	21 (60.0%)

a Other = laundress, manicurist, store owner, informal recycling.

b 4Ps = *Pantawid Pamilyang Pilipino Program* (national conditional cash transfer program).

In addition to the 35 female participants, 15 health care providers from 7 *barangays* were also interviewed. The participants included *barangay* health workers (BHWs) (n = 5), *barangay* nutrition scholars (BNSs) (n = 2), midwives (n = 4), physicians (n = 2), a nurse (n = 1), and a *barangay* health official (n = 1).

### Approachability and the ability to perceive

Participants reported that their main sources of health information came from family members, neighbors, and *barangay* health workers (BHWs). In particular, BHWs were often responsible for connecting individuals to available health services and providing information through their community outreach work. Trusting relationships between BHWs and community members were reported to be channels for the dissemination of health information, which subsequently informed community members of available resources. For instance, a woman from the youngest cohort (18-30 years) was aware of the immunization schedule at the local Rural Health Unit because she had “a booklet that has a record of the vaccinations and [the date of] the next visit. . .[it was provided] by the BHW.”

When confronted with minor symptoms such as fever, cough, body pains, or headache, all women reported they did not seek care at a health facility, but rather purchased over-the-counter medication from a *sari-sari* store (ie, convenience or corner store) or sought remedies from traditional healers (eg, *helots*). A woman from the oldest cohort (60 years and older) explained that individuals in her community were reluctant to seek care at health facilities due to a lack of financial resources. She explained, “if you are feeling bad that’s when you rush to the hospital but unless it’s. . .bad then you don’t have to go to the hospital because you don’t have money.” Several women indicated that over-the-counter medicines and traditional care were more affordable and convenient than formal health care due to transportation costs and other indirect costs associated with public health care. Moreover, their geographic proximity to and trusting relationships with traditional healers relative to public health care providers were reported to motivate care seeking from traditional healers.

### Acceptability and the ability to seek

Women used public and private health care facilities for more serious acute, chronic, and emergency health concerns. Public health care facilities were mainly used when participants perceived that the health concern could not be treated with over-the-counter medication, but lacked financial resources to access private care. A woman from the oldest age cohort (60 years and older) recounted an experience when one of her granddaughters was sick and explained, “if we have the money, we would prefer to go to a private doctor. . .because we are poor. . .then we just have the public.” In contrast, women gravitated toward accessing care at private clinics and hospitals if they perceived their health concern to be an emergency (eg, tuberculosis, chest pains, fainting) and they had sufficient financial resources. Private health services were preferred by some women due to the perception that private health care was more efficient and higher quality than public health care. For example, a participant from the youngest age cohort (18-30 years) explained that for her goiter, “I prefer private [care] because I [get] checked thoroughly. . .and I have not been checked thoroughly in the public [health facility].”

Younger women commonly reported experiences of seeking prenatal care or antenatal care or care for their children at both public and private facilities. In recounting experiences of seeking care for their children, some younger women described instances where they delayed care for themselves in order to prioritize the health of their child. As a woman from the youngest age cohort (18-30 years) explained:

I have [previously] been to a check-up [for my toxic goiter], but when I got pregnant with my youngest [child] I haven’t been able to have a checkup. . .Two years ago [was] when I had my last check-up [for the goiter] but I have [more recently] gone to the [nearest *Barangay* Health Station] for vaccines for the youngest [child].

In contrast, older women frequently reported age-related health issues (eg, hypertension, diabetes, and cardiovascular disease) and more commonly described experiences of seeking care for themselves.

### Availability, accommodation, and the ability to reach

Transportation was a consistent barrier to accessing health care at both public and private health care facilities, especially for women who lived in rural and peri-urban *barangays*. For example, a woman from the second youngest cohort (31-45 years) stated, “it is hard to find transportation . . .there’s actually [a] scheduled tricycle so after 9:00 [in the morning] there are no more tricycles to serve you so you need to wait for 2:00 [in the afternoon].” Transportation costs were also a challenge for most participants, and these costs prevented some women from reaching health care facilities. In addition to these challenges, some participants, especially younger mothers, reported that they had trouble finding childcare support when they needed to access a health care facility. If women could not find childcare support, their children often accompanied them to the health care facility, meaning that these women incurred higher transportation costs.

Health care provider insights supported these experiences reported by participants, as most providers recognized that indirect costs (eg, transportation costs) and the geographic distance to health facilities discouraged individuals experiencing poverty from seeking care. Health care providers also mentioned that a lack of stable work or precarious employment (eg, sugar cane farming), in addition to a lack of childcare, might create additional barriers to accessing health care. As a physician explained, “they will not come if they are too busy earning their money. . . I will ask them how come they [wait] this long to come to see the doctor and [they say] ‘I have no money’.” Due to these indirect and opportunity costs, several health care providers acknowledged that patients would often seek out geographically closer traditional health care or over-the-counter medications instead of accessing care at a formal health care facility. In some cases, the BHWs who were interviewed reported paying out-of-pocket to cover transportation costs to health facilities for some community members experiencing poverty.

Some women reported that they had assistance from their social networks (eg, family members, community members, or employers) to cover transportation costs, to provide childcare support, and to provide other non-financial supports when they experienced a health problem. Young women reported smaller perceived social networks, as most stated that they only had financial support from their parents or employers. As indicated above, women from the youngest age cohort were less likely to be enrolled in social protection programs (eg, 4Ps) that could provide additional financial resources in the case of a health problem. In contrast, some women from the second oldest cohort (age 46-59 years) had their older children offer to pay for transportation costs. For example, when a participant from the second oldest cohort (age 46-59 years) was asked who she could reach out to if she required assistance for a health problem, she stated “when I need help, I usually go first to my relatives and family within the community. . .my children gave me money and food and fruits as well.” Overall, older women reported a wider range of social connections compared to younger women, and more frequently mentioned their family members, communities, and social service agencies as sources of support to facilitate health care access.

Rural health units and public hospitals were the most accessed health facilities among participants and their families. Most women described quality issues at public health care facilities which included lengthy wait times and a shortage of available staff and resources. The anticipated long wait times prompted some women and their partners to take time off work to claim an early spot in the queue. However, the experience of lengthy wait times was not shared among all women, as a participant over the age of 60 explained that she was prioritized at a Rural Health Unit due to her age. In addition, a few women reported a shortage of health care staff and medical resources at some facilities, which negatively impacted their experience of health care utilization and resulted in them having to seek medical care elsewhere. These experiences prevented women from receiving timely care for themselves and their family members. These experiences were supported by the reflections of BHWs, who described the resource constraints (eg, medication shortages) they faced at their facilities and how these constraints contributed to an inability to accommodate all patient needs. In addition, some BHWs commented on how demand for health services was high and that they needed to work overtime and on weekends to meet patient demand.

Despite these quality concerns, several participants reported that their interactions with health care providers at public health care facilities were generally positive. However, other participants felt neglected and shared experiences of discrimination due to their socioeconomic status at public health care facilities. For example, a woman from the second oldest age cohort (age 46-59 years) expressed, “if you are very poor, poorest of the poor in the public [health facility] you do not get attention. . . so I like private [health facility] if I have money.” Thus, the quality concerns with public facilities and poor experiences with public health care providers prompted some individuals to favor private health care options, which subsequently increased their health care costs. Women who utilized private health care facilities reported faster assistance from the staff, attentive health care providers, and improved availability of medications.

### Affordability and the ability to pay

Participants with PhilHealth coverage mentioned that they used PhilHealth to settle all or a portion of their health care costs at public health care facilities. Several participants described scenarios whereby they were able to use PhilHealth to cover a portion of their public hospital stay, consultation with a physician, or diagnostic tests; however, the usage of PhilHealth appeared to be inconsistent among some participants who expressed uncertainty around what precisely was covered by the insurance under different circumstances. In addition, some women highlighted experiencing unexpected indirect costs such as the cost of food at a health facility during multi-day stays.

Following a preliminary diagnosis for a health problem, participants were often provided with a prescription for medication in addition to requisitions for further diagnostic tests (eg, blood work, medical imaging). Despite a few participants explaining that medications and diagnostic tests for certain health problems were covered by PhilHealth, other participants indicated that they had to pay out-of-pocket to fill prescriptions or for further diagnostic tests. In these cases, many participants opted to fill the prescription and forgo diagnostic tests because filling the prescription was the more affordable option. However, a woman in the second youngest age cohort (age 31-45 years) shared an experience where she could not afford to obtain a biopsy for her cyst. Consequently, health care providers could not determine her diagnosis, and the woman explained, “they did not prescribe any medicine for me to take because they do not know.” Additionally, some women stressed that they could not afford the medication they were prescribed.

In terms of direct health care costs, several health care providers explained that they ensured their patients were aware of government agencies and programs that could reduce or eliminate health care expenses. Some health care providers, especially private physicians, mentioned that they would also partner with local non-governmental organizations to provide free care to individuals experiencing poverty.

When PhilHealth coverage was insufficient, not accepted, or nonexistent, participants often approached their family members for financial support. Women in the youngest cohort (18-30 years) only reported reaching out to their relatives (eg, parents and siblings) for financial support to cover health care expenses including laboratory tests, diagnostic imaging, and medication. A few women in the youngest age cohort were reluctant to borrow money from their families as they did not want to appear to be a burden to their families. Consequently, these women often incurred loans or were required to save up money for anticipated health care expenses. In contrast, women in the other 3 age cohorts frequently received financial support from their family members, with some women explaining that they were not required to repay loans provided by family members. Some older women (46-59 years old; 60 years and older) also reported receiving remittances from their adult children who had migrated for work either within Negros Occidental or elsewhere in the Philippines.

In addition, most older women (46-59 years old; 60 years and older) were able to leverage their connections with local politicians and governmental agencies (eg, Department of Social Welfare and Development) to subsidize their health care costs. For example, several participants mentioned requesting financial assistance from municipal leaders when they experienced a large or emergency health expense. The older women (ages 46-59 years; 60 years and older) who had access to this financial support reported that they were able to seek appropriate treatment, adhere to treatment, and attend follow-up appointments. These women also explained that their existing social networks (eg, family members, neighbors) were critical in informing them about these sources of financial support to cover health care expenses.

### Appropriateness and the ability to engage

Following health care access and use, outstanding medical bills were a consistent concern raised in interviews among participants both with and without PhilHealth coverage. In particular, the ongoing burden of loans from health care expenses was highlighted among women in the youngest age cohort as a key consequence of health care access and use. Loans profoundly impacted the ability of these women to purchase necessities. Consequently, many young women explained that they relied on food donations from family members or loans from the local *sari-sari* store (ie, convenience or corner store) to meet the food needs of their families.

A few women explained that if future health emergencies arose, members within their social networks would be willing to provide financial assistance and food. Building a sense of reciprocity within social networks was mentioned as an important element to enhance the confidence among participants that they would be well-supported in case of a health emergency. For example, a participant from the second oldest age group (46-59 years) described her bond with her social network by explaining, “the feeling is mutual: if I need something, and they have it, they can extend help. For their family, as well: if I have something and they need help, I can extend the same.” This assurance of reciprocity in the case of a health emergency was a reported source of security for some participants.

After discussing various health care experiences, most participants described how their degree of satisfaction with a particular health care encounter was influenced by whether or not the encounter resolved their health problem. Efficiency in health care delivery was also highlighted by participants across age cohorts as a factor that shaped their overall satisfaction with specific health care access experiences. Importantly, satisfaction with health care experiences ultimately influenced attitudes toward and trust in specific health facilities, in addition to perceptions surrounding public and private health care. Moreover, participants indicated that satisfaction with previous health care experiences subsequently informed future health care decision making and health seeking behavior.

## Discussion

In this study, gender, age, and socioeconomic status influenced health care access and prevented some women from accessing timely care, with younger women more likely to delay seeking health care than older women. These findings align with related studies from the Philippines that report how younger women delay seeking health care as they prioritize their children’s health, have caregiving responsibilities, experience difficulties finding childcare, and, as caregivers, are deterred by the household financial burden associated with health care.^26,27,46^ Similarly, a study in Davao City, Philippines, found that most adult-child caregivers were, indeed, women living with their elderly parents—a gender-based role that can affect health care access and use.^23^ Evidently, within the Philippine context, gender can intersect with other individual, household, and structural level factors (such as perception of risk, affordability of services) to produce and amplify barriers to health care access and use among women experiencing poverty.

Access to quality health care services also depends on the availability of health care providers, resources, and infrastructure at the health systems level.^33^ In this study, appointment delays, lengthy wait times, and a shortage of health care providers were reported challenges that participants encountered when using public facilities. Long waiting times, in particular, can be considered an indicator of poorly distributed staff and equipment, as service demand can overwhelm available supply.^15^ As demonstrated through this study and elsewhere in South and South East Asia, an individual’s perception of the relatively poor quality of health care can also deter health care access and use, further exacerbating existing health care access barriers among women experiencing poverty.^47-49^

This study indicates that social networks may alleviate some of these barriers through building trust in health care services and facilitating financial and non-financial supports. Prior research in the Philippines has similarly explored these connections between social networks and health. For example, Lau et al^7^ found that family satisfaction and trust among individuals suspected of tuberculosis infection was associated with an increased likelihood of attending a Rural Health Unit to obtain tuberculosis testing. Social networks may also encompass individuals outside of the family unit. For instance, community-level factors such as religious beliefs and education, mediated through relationships, have been shown to impact individual reproductive and maternal health outcomes in the Philippines.^2^ As demonstrated in our study, social networks may also be communication channels for dissemination of information about the availability of health services, sources of financial support for health expenses, and positive health behaviors^50^—an important benefit, as formal health communication platforms can be limited in some Philippine contexts.^51^ Women also reported receiving health information from BHWs (ie, community health workers), a role within community social networks that may be increasingly important to effectively implement UHC.^52^

This study also demonstrated that gender norms and age can interact with social networks to influence experiences accessing health care services among women experiencing poverty. Due to the need for antenatal care, women in the youngest age cohort were among the most vulnerable to out-of-pocket health care expenditures. Similarly, women in the oldest age cohort required care for age-related health concerns; however, older women were able to leverage more social and financial support through their social networks compared to younger women. These findings illustrate that life stage may affect the size and strength of social networks among women experiencing poverty in the Philippines. Further, opportunities may exist for public health and health care practitioners to utilize social networks as channels for health information, outreach, and interventions, recognizing their importance for women of varying ages and life stages.

UHC policies have the potential to eliminate many direct health care costs, thus encouraging health care access and use among populations experiencing poverty.^53^ However, other potential indirect costs, such as transportation to health facilities, costs associated with long-term illnesses, and opportunity costs can still be significant deterrents to accessing care.^54,55^ Our findings identified that indirect health care costs can be significant barriers to health care access and use among women with and without PhilHealth coverage. A recent study described these expenses as “hidden” costs that continue to influence health-seeking behavior post-UHC legislation in the Philippines.^24^ A similar study demonstrated how PhilHealth coverage and related health care usage can vary contextually. Specifically, women with PhilHealth coverage from rural communities were found to only access health care facilities to give birth, but not for other health challenges.^17^ Thus, the elimination of direct health care costs does not necessarily translate into improved health care access and use.

In this study, health care access and use among women experiencing poverty was not only influenced by affordability, but also by perceived service quality, trust, and available health information. This finding is in line with prior literature identifying the importance of improving the quality of health care services alongside the expansion of PhilHealth coverage.^56^ Given their role in facilitating health care access and use for women, social networks may be an important mode of informal support to complement formal policy measures (eg, UHC), particularly for women experiencing poverty. As information channels, social networks may be leveraged for the provision of information regarding UHC and PhilHealth coverage and how programs are administered.

**Figure 1.**
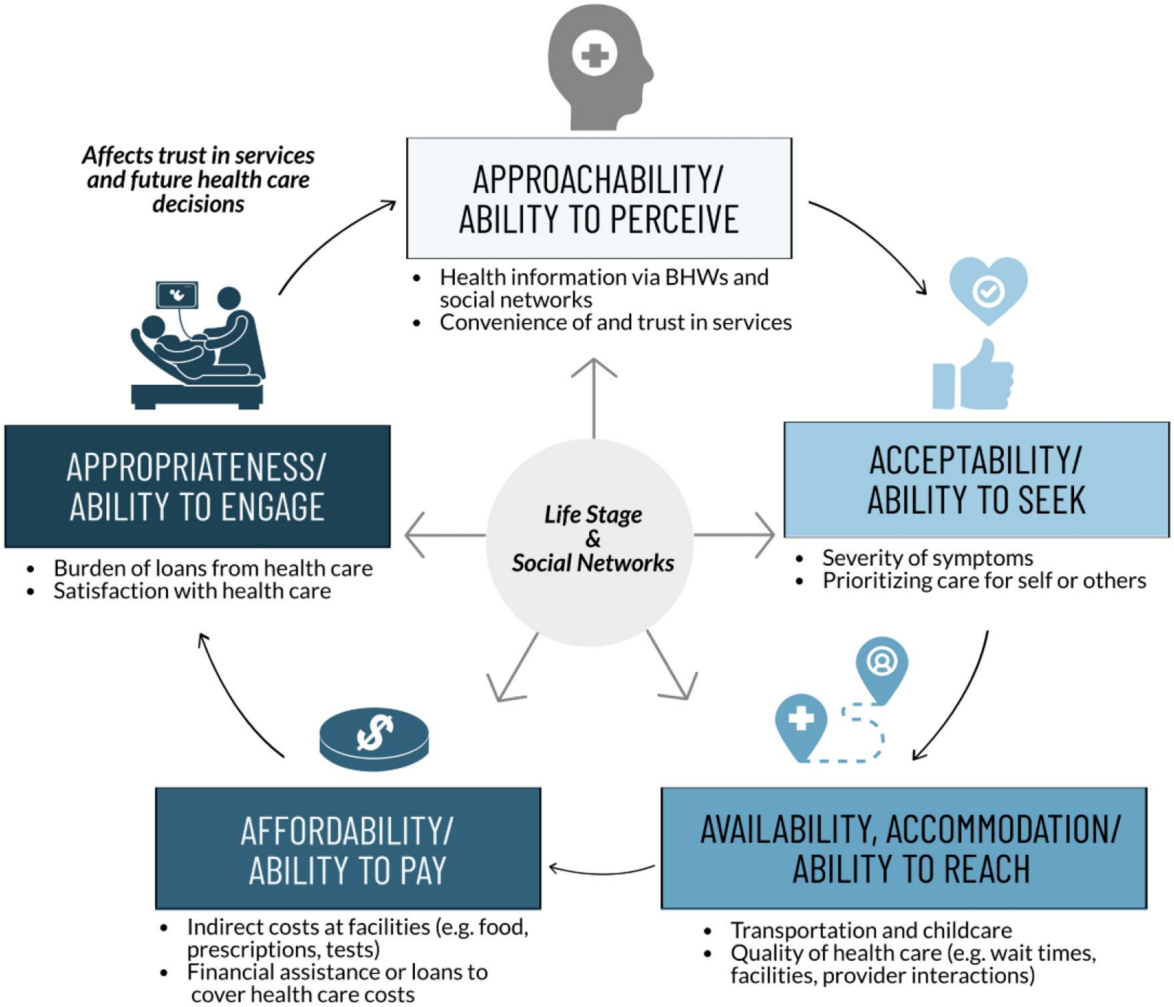
Mapping study results to elements of the Patient-Centred Access to Health Care (PCAHC) framework. Women experiencing poverty in the Philippines identified a cyclical—rather than linear—pathway of health care access and use. Barriers and facilitators to health care access and usage related to key dimensions of the PCAHC framework, including Approachability, Acceptability, Availability and Accommodation, Affordability, and Appropriateness. Life stage and social networks were cross-cutting determinants of health care access and use along the health care access pathway.

This study also provides important theoretical contributions in relation to the application of the PCAHC framework in examining health access and use in low resource settings. First, this study combines insights from both participants/patients and health care providers. Thus, this study contributes to the limited existing literature of how the PCAHC framework can be applied in cases where data is collected from both populations.^34^ Second, this study explicitly considers how life stage and social networks are cross-cutting themes and interact with each dimension of the PCAHC framework. This approach recognizes that health care access pathways are inherently shaped and informed by life stage and social networks, and that consideration of these factors cannot be isolated to a single dimension of the PCAHC framework. Finally, this study illustrates that health care access and use for women experiencing poverty in this context often follows a cyclical pathway, with previous health care experiences informing subsequent health seeking behavior (see Figure 1). The PCAHC framework has traditionally been presented as a linear model; however, we see an opportunity to more explicitly consider the cyclical nature of health care access and health seeking behavior through the PCAHC framework, especially among groups who have experienced discrimination or marginalization in health care settings.

### Limitations

This study has several limitations. First, participants in this study were not necessarily representative of the broader income-poor population in the research context. Participants were recruited according to their involvement in ICM programming and had similar demographic characteristics. In contrast, other individuals experiencing poverty in and around Bacolod City may not have access to programs provided by non-governmental organizations and, thus, may have additional or heightened barriers to health care access and use. Second, the health system in the Philippines is highly decentralized, meaning that the quality and delivery of health services can differ across settings. Thus, participant experiences shared through this study were influenced by their specific context and may not be a reflection of health system dynamics elsewhere in the Philippines. Nevertheless, our study highlights the potential role of social networks in facilitating health care access and use in a changing health policy landscape—a finding that is likely to have broader relevance throughout the Philippines.

### Future research

Findings from this study can provide a foundation for future research in several areas. First, as UHC roll-out continues in the Philippines and in other countries, further research is needed to understand how this roll-out influences, and perhaps alters, health care access and use among individuals and households experiencing poverty. Second, further research could more fully investigate the potential synergies between social networks and health policy measures such as UHC to examine the extent to which these synergies translate into reduced barriers to health care access and use among individuals and households experiencing poverty. Finally, there is an opportunity to test and evaluate different interventions to support and strengthen social networks among individuals experiencing poverty in low resource settings. In particular, research is needed to determine the effectiveness of these interventions in facilitating health care access and use, in addition to the feasibility of scaling these interventions across settings.

## Conclusions

In the context of recent UHC legislation in the Philippines, this study explored health care access and use among women experiencing poverty in and around Bacolod City, Negros Occidental. We found that although recent health reforms in the Philippines have attempted to alleviate the financial burden at the point of care for individuals experiencing poverty, women relied on their social networks for financial support when health insurance coverage did not adequately address the direct and indirect costs along the healthcare access pathway. In addition, these findings demonstrated the importance of social networks in mitigating the social and financial burdens associated with accessing health care services among women at different life stages in this context. These findings suggest that supporting and strengthening social networks among women experiencing poverty can complement existing efforts to implement UHC in the Philippines.
